# Repurposing Benzbromarone for Familial Amyloid Polyneuropathy: A New Transthyretin Tetramer Stabilizer

**DOI:** 10.3390/ijms21197166

**Published:** 2020-09-28

**Authors:** Ellen Y. Cotrina, Ângela Oliveira, José Pedro Leite, Jordi Llop, Luis Gales, Jordi Quintana, Isabel Cardoso, Gemma Arsequell

**Affiliations:** 1 Institut de Química Avançada de Catalunya (I.Q.A.C.-C.S.I.C.), 08034 Barcelona, Spain; ellen.cotrina@iqac.csic.es; 2 IBMC—Instituto de Biologia Molecular e Celular, 4200-135 Porto, Portugal; angela.d.a.oliveira@gmail.com (Â.O.); jose.leite@ibmc.up.pt (J.P.L.); lgales@ibmc.up.pt (L.G.); 3 i3S—Instituto de Investigação e Inovação em Saúde, Universidade do Porto, 4200-135 Porto, Portugal; 4 Instituto de Ciências Biomédicas Abel Salazar (ICBAS), 4050-013 Porto, Portugal; 5 CIC biomaGUNE, Basque Research and Technology Alliance (BRTA), 20014 San Sebastian, Spain; jllop@cicbiomagune.es; 6 Research Programme on Biomedical Informatics, Universitat Pompeu Fabra (UPF-IMIM), 08003 Barcelona, Spain; jordiramon.quintana@upf.edu

**Keywords:** benzbromarone, drug, transthyretin tetramer stabilizer, transthyretin, drug repurposing, native kinetic stabilization, dibromophenol scaffold

## Abstract

Transthyretin (TTR) is a homotetrameric protein involved in human amyloidosis, including familial amyloid polyneuropathy (FAP). Discovering small-molecule stabilizers of the TTR tetramer is a therapeutic strategy for these diseases. Tafamidis, the only approved drug for FAP treatment, is not effective for all patients. Herein, we discovered that benzbromarone (BBM), a uricosuric drug, is an effective TTR stabilizer and inhibitor against TTR amyloid fibril formation. BBM rendered TTR more resistant to urea denaturation, similarly to iododiflunisal (IDIF), a very potent TTR stabilizer. BBM competes with thyroxine for binding in the TTR central channel, with an IC_50_ similar to IDIF and tafamidis. Results obtained by isothermal titration calorimetry (ITC) demonstrated that BBM binds TTR with an affinity similar to IDIF, tolcapone and tafamidis, confirming BBM as a potent binder of TTR. The crystal structure of the BBM-TTR complex shows two molecules binding deeply in the thyroxine binding channel, forming strong intermonomer hydrogen bonds and increasing the stability of the TTR tetramer. Finally, kinetic analysis of the ability of BBM to inhibit TTR fibrillogenesis at acidic pH and comparison with other stabilizers revealed that benzbromarone is a potent inhibitor of TTR amyloidogenesis, adding a new interesting scaffold for drug design of TTR stabilizers.

## 1. Introduction

**Figure 1:**
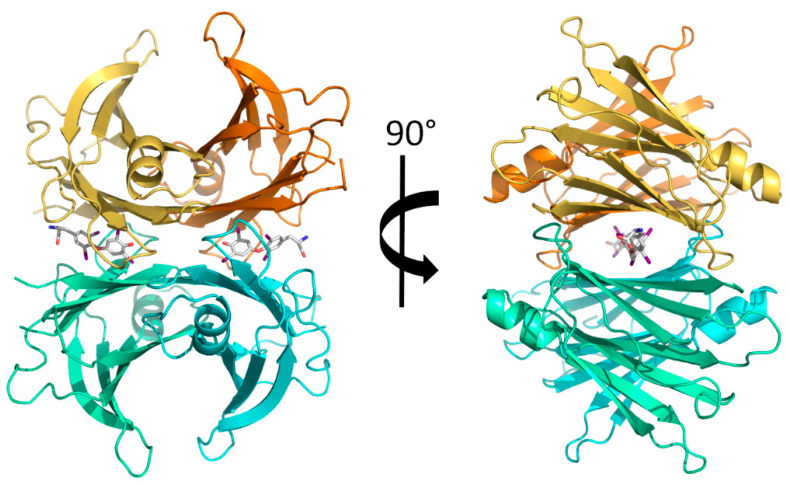
Crystal structure of transthyretin (TTR) bound to the natural ligand thyroxine (T4, PDB ID: 2ROX). The functional TTR homotetramer is assembled by two TTR dimers, AB in orange and A’B’ in cyan.

Transthyretin (TTR) is a protein of 127 amino acids that self-assembles as a 55 kDa homotetramer that is secreted into the bloodstream by the liver [1,2] and into the cerebrospinal fluid (CSF) by the choroid plexus [3]. TTR transports holo-retinol-binding protein in the blood, forming a macromolecular complex [4,5]. The protein acts as a backup carrier of thyroxine (T4) in serum (crystal structure in Figure 1), where the principal transporters are thyroid-binding globulin (TBG) and albumin, while being the primary carrier of T4 in CSF. The T4-binding sites at the weaker of the two dimer–dimer interfaces of TTR are largely unoccupied in human blood and CSF [6]. TTR likely has additional unknown functions, especially in the brain [7]. In fact, TTR is also known as a neuroprotective protein in Alzheimer’s disease (AD) [8,9,10].

TTR has been the subject of intensive research in the last 30 years because of its pathogenic properties. Transthyretin (TTR) amyloidoses are a group of systemic degenerative diseases involving multiple organ systems and are caused by TTR aggregation [11], such as familial amyloid polyneuropathy (FAP) [12], familial amyloid cardiomyopathy (FAC) [13], senile systemic amyloidosis [14] and leptomeningeal amyloidosis [15]. 

Extensive efforts have been made in the past to investigate the aggregation pathway of TTR and to discover small-molecule compounds that stabilize the natively folded, non-amyloidogenic, tetrameric structure of the protein by docking on its T4 binding pocket and preventing its aggregation. This pharmacological discovery was referred to as the native kinetic stabilization strategy [16]. The synthetic chemistry efforts ultimately yielded >1000 small-molecule TTR kinetic stabilizers that are potent aggregation inhibitors.

Currently, more than 400 crystallographic structures of TTR are deposited in the Protein Data Bank (http://www.rcsb.org) [17], most of them in complex with small-molecule ligands. This comprehensive work has allowed a full characterization of the TTR binding sites [18,19,20]. A relevant feature of these sites is the presence of three sets of symmetry-related depressions termed halogen-binding pockets (HBPs: HBP1 and HBP1’, HBP2 and HBP2’ and HBP3 and HBP3’), which accommodate the iodine atoms of the thyroid hormones in their complexes with TTR [21]. The innermost pockets are HBP3 and HBP3’ and the outermost HBP1 and HBP1’.

**Scheme 1:**
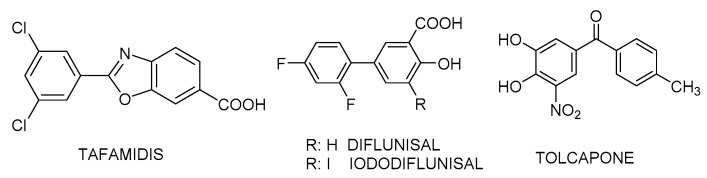
Chemical structures of TTR tetramer stabilizers: the orphan drug tafamidis, the Non-steroidal anti-inflammatory drug (NSAID) diflunisal, the repurposed drug tolcapone for Familial amyloid polyneuropathy (FAP) and our lead compound iododiflunisal (IDIF).

To date, only tafamidis (registered as Vyndaqel^®^; Scheme 1) [22], a benzoxazole derivative fashioned by structure-based drug design, and a repurposed drug, the non-steroidal anti-inflammatory drug (NSAID) diflunisal (Scheme 1) [23], have reached approval for clinical use. The drug tolcapone (Scheme 1), an orally active catechol-*O*-methyltransferase (COMT) inhibitor authorized in the United States and Europe as an adjunct to levodopa and carbidopa for the treatment of Parkinson’s disease, has also been repurposed for FAP [24].

The drug discovery effort focused on TTR amyloid diseases continues, exemplified by the discovery of the small-molecule stabilizer AG10, now in a phase 3 trial (NCT03860935) [25].

One of our contributions to this ongoing drug discovery effort has been the preclinical development of iododiflunisal (IDIF, Scheme 1) [26,27,28] an iodinated derivative of the NSAID diflunisal.

**Scheme 2:**
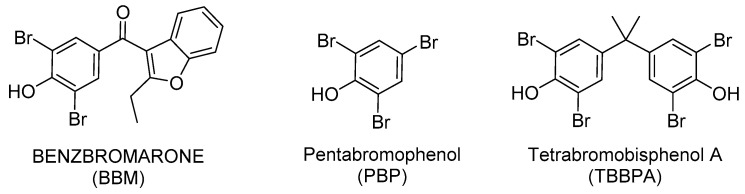
Chemical structures of the small-molecule compounds sharing a dibromophenol scaffold: the drug benzbromarone (BBM) and the TTR ligands pentabromophenol (PBP, PDB ID: IE3F) and tetrabromobisphenol A (TBBPA, PDB ID: 5HJG).

Interestingly, a high percentage of the TTR T4-binding tetramer stabilizers reported in the Protein Data Bank [17] are halogenated phenol compounds (Supporting Information, Table S2). Some brominated ligands of TTR are pentabromophenol (PBP; Scheme 2) [29] and the flame retardant tetrabromobisphenol A (TBBPA; Scheme 2) [30]. TBBPA binds in the thyroxine-binding pocket, with bromines occupying two of the three halogen-binding sites. According to the authors, this molecule represents an interesting scaffold. Moreover, the effect of the halogenation on the biological activity of previously reported TTR tetramer stabilizers has also been assayed to provide mechanistic insights into their binding properties [31,32,33].

The drug benzbromarone (BBM; Scheme 2) shares with TBBPA an interesting dibromophenol scaffold for drug design, being a candidate drug for repurposing purposes [34]. BBM has been used for more than 30 years to control hyperuricemia and gout [35,36]. Interest in this drug was found in other recent research studies. BBM was reported to be an effective inhibitor of amylin aggregation [37]. BBM was shown to inhibit retinol-dependent RBP4-TTR interaction for treatment of age-related macular degeneration [38].

In the present work, we investigated the potential of the registered drug BBM as a TTR tetramer stabilizer. To this end, we report a comparative in vitro and ex vivo study of the binding affinity of BBM to TTR; a comparative ITC analysis of the thermodynamic profile of the TTR/BBM with other stabilizers; an X-ray analysis of the molecular details of the binary interaction of BBM with TTR resulting in a strong TTR tetramer stabilization; and a comparative kinetic analysis of TTR fibrillogenesis in the presence of BBM with other stabilizers.

## 2. Results and Discussion

### 2.1. Benzbromarone (BBM) Stabilizes the TTR Tetrameric Fold

To determine if BBM fits the native kinetic stabilization strategy, we investigated the effect of BBM in TTR stability by measuring TTR resistance to urea denaturation, as previously described [39]. The extent of denaturation of the TTR tetramer was evaluated by measuring how much folded TTR (tetramer, trimer and dimer) and monomer remained after urea treatment. We compared the effect of BBM to iododiflunisal (IDIF), known to be a potent TTR stabilizer, used here as a positive control, and also to sulfaquinoxaline, a compound that does not stabilize TTR and used here as a negative control.

**Figure 2:**
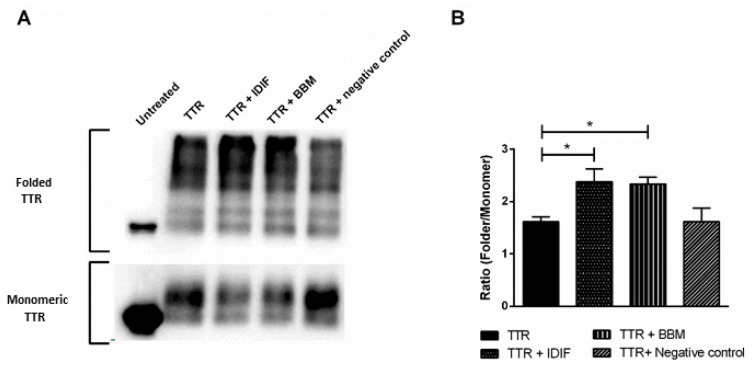
TTR resistance to urea denaturation. (**A**) TTR, alone or pre-incubated with different compounds, was subjected to urea denaturation, as described, and the generated species were detected by western blot. Incubation with iododiflunisal (IDIF) and benzbromarone (BBM) resulted in increased folded TTR, as compared to the negative control and TTR alone. (**B**) Quantification of results from three independent experiments. Error bars represent SEM. * *p* < 0.05.

As depicted in Figure 2, pre-incubation of TTR with BBM increased the resistance of TTR to urea denaturation, as compared to TTR alone. The level of TTR stabilization provided by BBM was comparable to the one by IDIF. As expected, the compound sulfaquinoxaline, used as a negative control, did not change the levels of folded/monomeric TTR, which were found to be similar to the ones in TTR incubated alone.

### 2.2. Benzbromarone (BBM) Binds TTR in the T4 Central Binding Pocket

**Figure 3:**
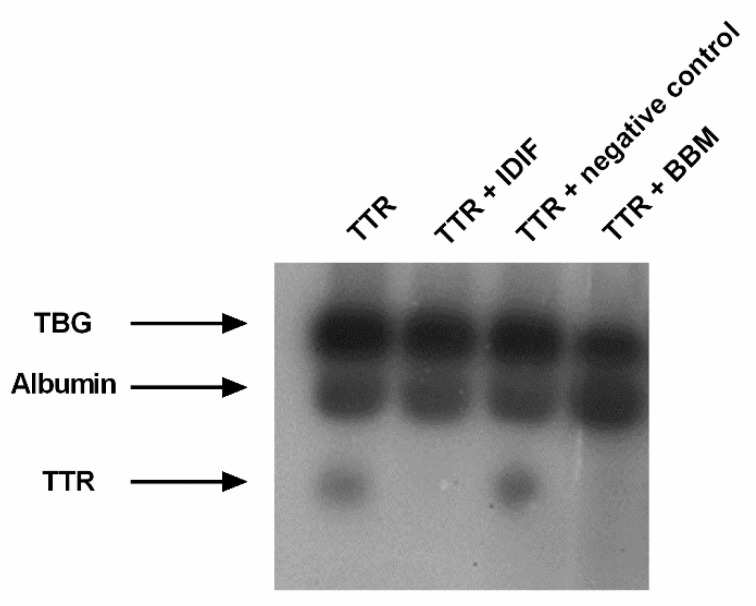
T4 displacement from TTR. Native gel electrophoresis of human plasma, incubated with ^125^I-T4 and competitor, showing that IDIF and BBM can compete with T4 for its binding site, whereas the negative control could not displace T4 from TTR. The migration of the different plasma T4-binding proteins is indicated.

We then investigated if the capacity of BBM to stabilize TTR was related to its binding in the T4-binding channel, as for the other TTR stabilizers mentioned. In this qualitative assay, we used human plasma as the source of TTR and measured the degree of ^125^I-T4 displacement by evaluating the decrease in the intensity of the TTR/^125^I-T4 band. As shown in Figure 3, BBM completely displaced T4 from TTR, similarly to IDIF. The negative control (sulfaquinoxaline) did not displace T4 from TTR and thus the intensity of the TTR/T4 band was similar to the one observed for TTR incubated in the absence of stabilizer.

As for the selectivity of BBM displacement of T4, with other proteins, BBM mainly displaced T4 from TTR, although it was possible to observe a small decrease in the intensity of the TBG/T4 bands and increase in the albumin/T4 bands (Figure 3).

**Figure 4:**
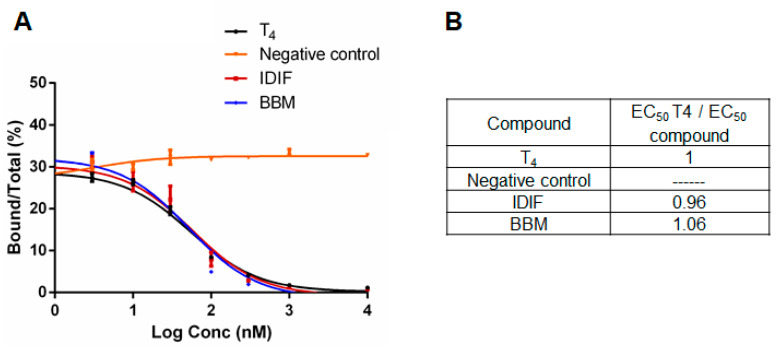
(**A**) Displacement of ^125^I-T4 from TTR by competition with the compounds IDIF and BBM, the natural ligand T4 is used as a positive control and sulfaquinoxaline is used as a negative control; (**B**) relative potency for the inhibition of binding of T4.

To characterize the TTR/BBM interaction, and since the T4 displacement gel approach is only qualitative, we quantified the interaction using competition assays as described in the Materials and Methods (Figure 4A), and calculated the EC50 for each compound. The relative potency for the inhibition of the binding of T4 was defined as the ratio of (EC50 T4)/(EC50 small molecule) (Figure 4B). The results demonstrated that BBM had a high relative inhibition potency (1.06) similar to that of IDIF (0.96), prompting BBM as a potent TTR stabilizer. The negative control did not compete with T4 for TTR binding.

### 2.3. Isothermal Titration Calorimetry (ITC) Studies of the Binary Interaction between TTR and the Small-Molecule Drug Benzbromarone (BBM). Comparison with other TTR Binary Interactions with Iododiflunisal (IDIF), Tolcapone and Tafamidis

ITC is one of the gold standard biophysical methods for the quantitative analysis of intermolecular interaction, providing the complete thermodynamic profile in terms of free energy (ΔG), enthalpy (ΔH), entropy (ΔS), binding constant (Kd) and stoichiometry (n) of the interaction from a single experiment [40]. The Kd for the binding of a stabilizer to TTR is represented by the change in the Gibbs free energy of binding (ΔG), where ΔG = ΔH − TΔS. This technique has been used previously to compare small-molecule TTR stabilizers, like the small-molecule AG10 with other TTR tetramer stabilizer drugs [41].

**Figure 5:**
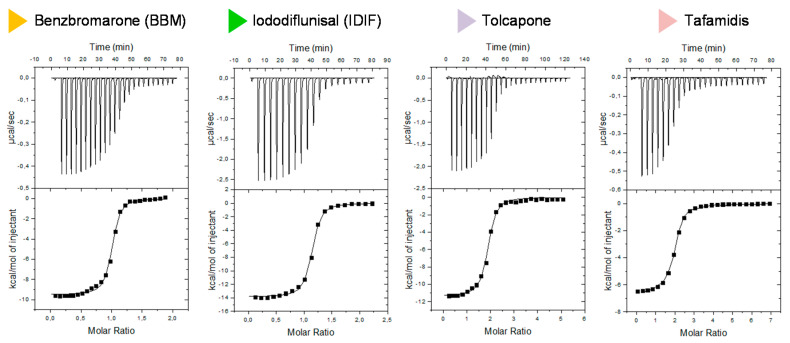
Thermograms of different complexes between TTR and small-molecule compounds: TTR + BBM, TTR + IDIF, TTR + tolcapone and TTR + tafamidis.

ITC studies were performed to characterize the thermodynamic profile of the BBM binding to TTR. In this work, we compared the thermodynamic signatures of the interactions of TTR with other stabilizers, our lead compound IDIF and the TTR tetramer stabilizer drugs tolcapone and tafamidis (Figure 5).

As it can be observed (Table 1), the binding stoichiometry number n is 1 both for BBM and for IDIF, whereas for tolcapone and tafamidis, n is 2. The Kd values reported in Table 1 were based on data fitted to an independent single-site binding model implemented in Origin 7.0 (OriginLab). Previous ITC studies for the binary complexes of tolcapone and tafamidis with TTR have used either a single model [42] or a two-site model to approximate binding to TTR [24,41].

The binding interactions for BBM with TTR are enthalpy driven (ΔH = −9.93 kcal/mol), similar to the binding interactions of our lead compound IDIF with TTR, but in the case of IDIF, the enthalpic contrition is higher (ΔH = −13.10 kcal/mol). BBM binds to TTR with a slightly unfavorable entropy contribution (TΔS = −0.02 kcal/mol) compared to the unfavorable entropy contribution (TΔS = −3.61 kcal/mol) of IDIF. BBM shows similar binding affinities to TTR in buffer (i.e., similar ΔG values) compared to IDIF, tolcapone and tafamidis. These ITC results confirm that BBM is a potent binder of TTR.

### 2.4. Crystal Structure of TTR in Complex with BBM (TTR:BBM Complex)

X-ray analysis was conducted to elucidate the molecular details of the interactions of BBM with TTR. Crystals of TTR in complex with BBM diffracted to a maximum of 1.35 Å (see Supporting Information Table S1).

**Figure 6:**
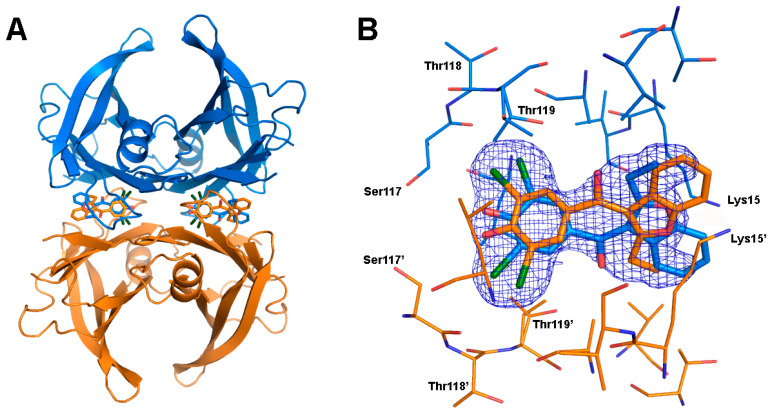
(**A**). Crystal structure of TTR in complex with benzbromarone (BBM). Monomers A and B and their symmetry-related pair (shown in light blue and brown) form a cavity in the dimer interface that can simultaneously accommodate two BBM molecules. (**B**) Close-up view of the BB’ binding site, showing the two symmetry-related positions of BBM. 2Fo-Fc map (blue mesh) contoured around BBM at 1.0 σ. Bromine atoms in green; oxygen in red; nitrogen in dark blue.

The two dimers that form TTR are related by a crystallographic 2-fold axis that runs along a central channel. The channel displays two hormone-binding sites, AA’ and BB’, with similar topology. BBM molecules bind deeply within the thyroxine binding pocket, with bromine atoms at the inner side of the cavity (Figure 6). In the crystal structure of the apo-TTR, Ser117 side chains face the cavities and hydrogen bonded to crystallographic water molecules. BBM binding induces the rotation of the side residues of serine 117 of all four monomers, prompting the formation of strong intermonomer hydrogen bonds which presumably increase the stability of the TTR tetramer.

**Figure 7:**
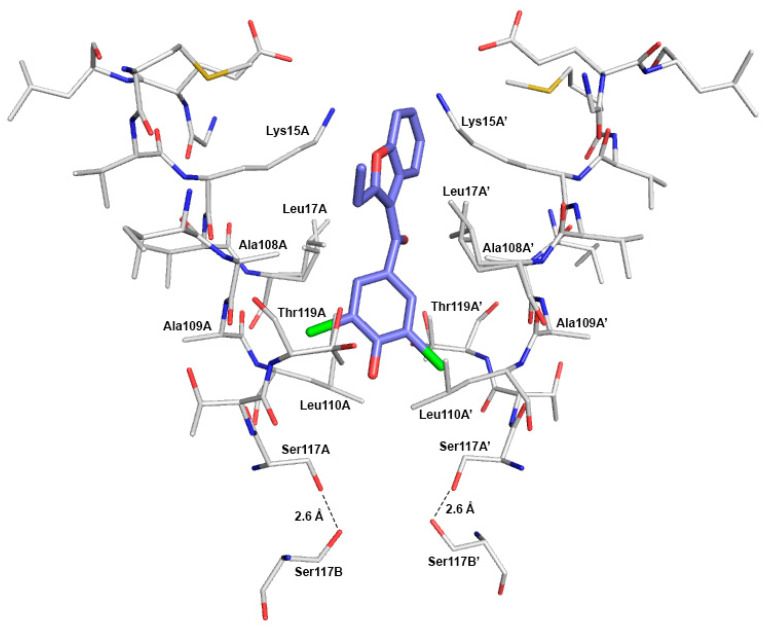
Close view of the interactions of benzbromarone (BBM) with the TTR AA’ binding site (carbon atoms in grey, nitrogen atoms in dark blue, oxygen in red, sulfur in dark yellow and bromine atoms of BBM in green).

Three pairs of symmetry-related binding pockets (HBP1 and HBP1’, HBP2 and HBP2’, HBP3 and HBP3’) are found within each hormone-binding pocket according to the positions of the iodine atoms of T4, when bound to TTR. BBM binds TTR in forward mode (Figure 7), with the bromine-substituted ring occupying the innermost part of the T_4_ binding site. The two bromine atoms are anchored in HBP3’, comprising the side chains of Ser117’, Leu110’, Thr119’ and Ala108’, and in HBP2, comprising residues Leu17, Ala108, Ala109 and Leu110, respectively. Ligand binding induces the rotation of the side chains of Ser117 residues of the four monomers, leading to the formation of strong intermonomer hydrogen bonds.

**Figure 8:**
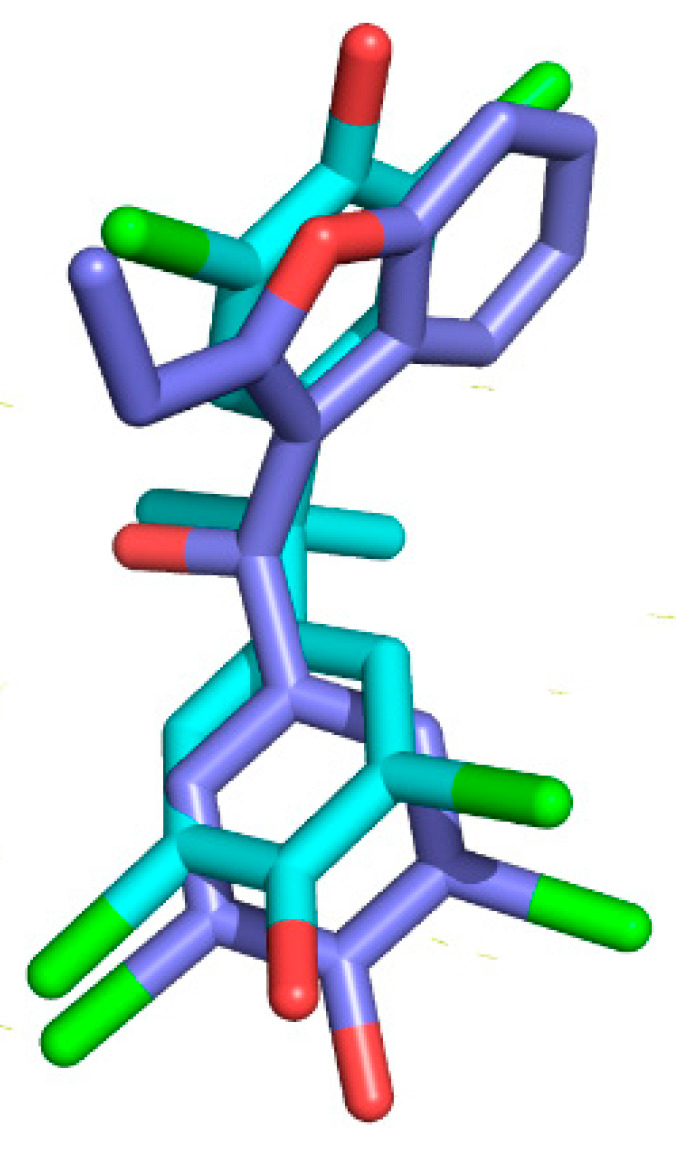
Close superposition of TTR AA’ binding site of the complexes of TTR with BBM and with TBBPA (PDB ID: 5HJG). Only the ligands are shown to highlight the similarities of their binding modes. The orientation of the protein binding site is similar to the one in Figure 7. BBM and TBBPA atom colors as in Figure 7 except for TBBPA carbon atoms shown in light blue.

The binding mode of BBM to TTR is very similar to the one of the abovementioned TBBPA. Nevertheless, superposition of the two crystals structures reveals that BMM binds slightly deeper than TBBPA (Figure 8).

### 2.5. Comparative Analysis of the Ability of BBM to Inhibit TTR Fibrillogenesis at Acidic pH

**Figure 9:**
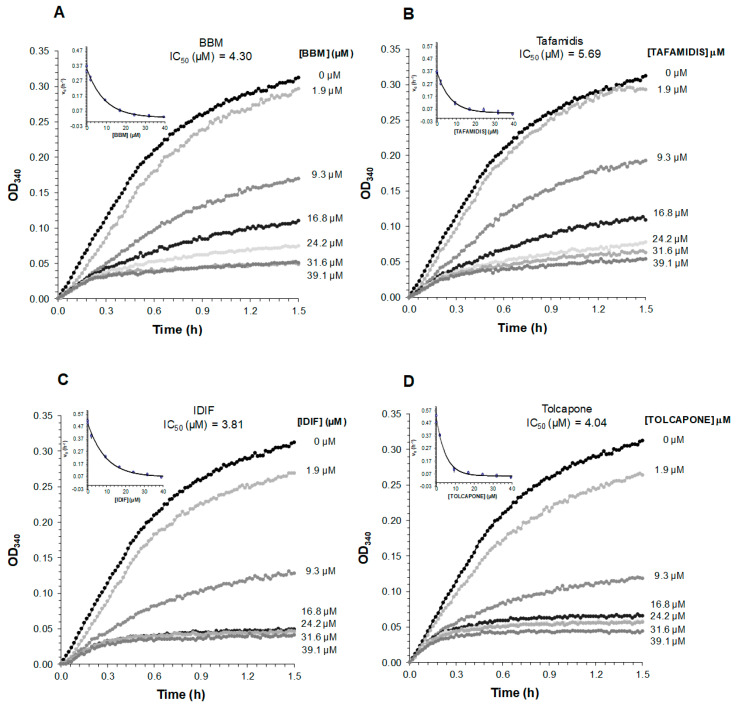
Time course of Y78F hTTR fibril formation at pH 4.2, 37 °C in the presence of different concentrations of: (**A**) BBM (inset: plot of initial rates of fibril formation (V0) vs. BBM concentration (Figure S1). Data were fitted to eq 1); (**B**) tafamidis (inset: plot of initial rates of fibril formation vs. tafamidis concentration) (Figure S2); (**C**) IDIF (inset: plot of initial rates of fibril formation vs. IDIF concentration) (Figure S4); and (**D**) tolcapone (inset: plot of initial rates of fibril formation vs. tolcapone concentration) (Figure S3).; for diflunisal (Figure S5); as monitored by absorbance at 340 nm at different concentrations. Experiments were performed in duplicate.

Finally, to investigate the impact of BBM on TTR aggregation, we determined the effect of BBM on the aggregation of the amyloidogenic Y78F hTTR mutant, by using our kinetic assay [43] that follows turbidimetry of TTR at 340 nm (Figure 9A), and performed a comparative analysis with other TTR stabilizers, such as tafamidis (Figure 9B), tolcapone (Figure 9D) and IDIF (Figure 9C).

Kinetic measurements showed that BBM efficiently prevents the aggregation of Y78F hTTR at low pH, with IC_50_ = 4.30 µM, in the same order as the IC_50_ for our small-molecule IDIF (IC_50_ = 3.81 µM) and the ones for the drugs tafamidis (IC_50_ = 5.69 µM) and tolcapone (IC_50_ = 4.05 µM).

## 3. Materials and Methods

### 3.1. Compounds

Tafamidis was synthesized in our laboratory according to reported procedures in the literature [44]. HEPES, *N*-(2-Hydroxyethyl) piperazine-*N*′-(2-ethanesulfonic acid); glycine; dimethyl sulfoxide (DMSO), the NSAID drug diflunisal (DIF) and the drug tolcapone were commercially available from Sigma-Aldrich (St. Louis, MO, USA). Iododiflunisal (IDIF) was prepared in our lab by iodination of the NSAID diflunisal, following our previously described procedures [28]. Purity of all compounds was proved to be ≥95% by means of analytical HPLC, NMR and UPLC-TOF-MS techniques.

### 3.2. TTR Production and Purification

Human recombinant wild-type TTR (wt TTR) and the mutant Y78F (Y78F hTTR) were produced in a bacterial expression system using *Escherichia coli* BL21 [45] and purified as previously described [46]. Briefly, after growing the bacteria, the protein was isolated and purified by preparative gel electrophoresis after ion exchange chromatography. Protein concentration was determined by the Bradford method (Bio-Rad, Hercules, CA, USA), using bovine serum albumin (BSA) as standard.

### 3.3. TTR Stability Assay

Recombinant wtTTR was incubated alone or in the presence of different compounds: IDIF as a reference positive control, the veterinary drug sulfaquinoxaline as a negative control, and the BBM drug at a molar ratio of 1:10 (TTR:drug) for 1 h at 37 °C. Then, urea was added at 6 M and samples were further incubated at 37 °C, overnight. The cross-linking reaction was performed by adding 2.5% glutaraldehyde for 4 min and then the reaction was quenched by adding 0.1% sodium borohydride. Samples were then run in a 13.5% acrylamide gel prepared with SDS, and transferred onto a nitrocellulose membrane (Amersham NC Protran^TM^ 0.2 μm Amersham GE Healthcare, Buckinghamshire, UK) using a wet system (Bio-Rad Criterion Blotter). The membranes were blocked for 1 h at RT with 5% nonfat dry milk (DM) in PBS containing 0.05% Tween-20 (PBS-T) and then incubated with primary antibody antihuman TTR (Dako; 1:1000 in 3% DM/PBS-T, Dako, Glostrup, Denmark). Then, washed membranes were incubated for 1 h at RT with sheep antirabbit immunoglobulins conjugated with horseradish peroxidase (Binding Site; 1:5000 in 3% DM/PBS-T). The blots were developed using Clarity^TM^ Western ECL substrate (Bio-Rad), and levels of folded (tetramer + trimer + dimer) and monomeric TTR were detected and visualized using a chemiluminescence detection system (ChemiDoc, Bio-Rad).

### 3.4. Thyroxine Binding Assays

Qualitative studies of the displacement of T4 from TTR were carried out by incubation of 5 μL of human plasma, with ^125^I-T4 (specific radioactivity ≈ 1200 μCi/μg; Perkin Elmer) in the presence of the different compounds (IDIF, BBM and sulfaquinoxaline) at a final concentration of 666 μM. Protein separation was carried in a native PAGE system using glycine/acetate buffer. The gel was dried and revealed using an X-ray film.

For the quantitative analysis, T_4_ binding competition assays based on a gel filtration procedure were used, as previously described [47]. Briefly, 30 nM human recombinant wtTTR was incubated with cold T4 or compound (IDIF, BBM and sulfaquinoxaline negative control) solutions of variable concentrations ranging from 0 to 1000 nM and with a constant amount of labeled ^125^I-T4 (~50,000 cpm). This solution was counted in a gamma spectrometer and incubated at 4 °C overnight. Protein-bound ^125^I-T4 and free ^125^I-T4 were separated by gel filtration through a 1 mL BioGel P6DG (Bio-Rad, Hercules, CA, USA) column. The bound fraction was eluted while free T4 was retained on the BioGel matrix. The eluate containing the bound T4 was collected and counted. Bound T4 was expressed as a percentage of total T4 added. The experiment was performed twice, and each assay was done in duplicate. Analysis of the binding data was performed with the GraphPad Prism program (version 5.0, San Diego, CA, USA) and data were expressed as the ratio of EC_50_ T4/EC_50_ compound.

### 3.5. Isothermal Titration Calorimetry (ITC) Studies

Isothermal titration calorimetry (ITC) measurements were carried out in a VP-ITC (MicroCal, LLC, Northampton, MA, USA). In a titration experiment, the ligand in the syringe is added in small aliquots to the TTR protein in the calorimeter cell, which is filled with an effective volume that is sensed calorimetrically. All solutions, either TTR protein or drugs, were prepared in 25 mM HEPES buffer, 10 mM glycine, pH 7.4 and 5% DMSO (final concentration). The concentration of TTR solution was 5–10 µM and 50–100 µM for ligand solutions. All solutions were filtered and degassed prior to usage. The TTR protein solution was injected over 20 or 30 times at a constant interval of 300 s with a 450 rpm rotating stirrer syringe into the sample cell. In the control experiment, the ligand was injected into the buffer in the sample cell to obtain the heat of dilution. The value of the heat of dilution was subtracted from the experimental result in the final analysis. The experiments were performed at 25 °C, reference power at 10. Titration data were analyzed by the evaluation software, MicroCal Origin, Version 7.0, provided by the manufacturer. Calorimetric data were plotted and fitted using the standard single-site binding model. The binding curves were fitted to the calculations of the parameters’ stoichiometry (n), dissociation constant (Kd) and the changes in the enthalpy (ΔH), entropy (ΔS) and Gibbs free energy (ΔG) during the complex formation. Each experiment was done three times.

### 3.6. Co-Crystallization

The wtTTR at 182 µM was incubated with BBM (molar ratio BBM/wtTTR = 13) at 4 °C for 16 h, in 10 mM HEPES buffer, pH 7.5. Crystals suitable for X-ray diffraction were obtained by the hanging-drop vapor-diffusion technique at 20 °C and were grown within 2 weeks by mixing 2 μL of the protein:BBM solution with 2 μL of reservoir solution. The reservoir solution contained 0.2 M acetate buffer pH 4.8, 2.2 M ammonium sulfate and 7% glycerol. Crystals were transferred to reservoir solutions containing increasing concentrations of glycerol (10–25%) and flash frozen in liquid nitrogen.

### 3.7. X-ray Diffraction Data Collection, Processing and Structure Refinement

X-ray diffraction data sets were collected using synchrotron radiation (λ = 0.979 Å) at the Proxima 1 beam line of the SOLEIL synchrotron (Paris, France). Preliminary data were measured in ESRF and ALBA synchrotrons. A summary of the data collection and refinement statistics is presented in Table S1 in the Supporting Information. Diffraction images were processed with the iMosflm software [48] and the scaling and merging of the reflections were performed using programs SCALA and TRUNCATE [49]. A random 5% sample of the reflection data was flagged for R-free calculations [50] during model building and refinement. Initial molecular replacement phases were generated with Phaser MR [51], using as the initial model one monomer of the complex TTR:IDIF (PDB ID: 1Y1D) [27] followed by refinement cycles performed with Refmac5 [49]. The final models were obtained after further cycles of manual model building and refinement, carried out with Coot [52] and PHENIX [53], respectively. Structural data were deposited in the Protein Data Bank (PDB) with PDB ID code 7ACU and figures of the protein model were generated with PyMol [54].

### 3.8. Kinetic Turbidity Assay

Protein (Y78F hTTR) stock: 4 mg/mL in 20 mM phosphate, 100 mM KCl, pH 7.6. Incubation buffer: 10 mM phosphate, 100 mM KCl, 1 mM EDTA, pH 7.6. Dilution buffer: 400 mM sodium acetate, 100 mM KCl, 1 mM EDTA, pH 4.2. Protocol for one compound: 20 µL of Y78F hTTR stock was dispensed into seven wells of a 96-well microplate. Different volumes of working inhibitor solution were added to give final concentrations ranging from 0 to 40 µM, and the final DMSO content of each well was adjusted to 5% by adding the corresponding volume of a H2O/DMSO (1:1) solution. Incubation buffer was then added up to a volume of 100 µL. The plate was incubated at 37 °C in a thermostated microplate reader with orbital shaking 15 s every minute for 30 min. A 100 µL portion of dilution buffer was dispensed to each well, and the mixture was incubated at 37 °C with shaking (15 s every min) in the microplate reader. Absorbance at 340 nm was monitored for 1.5 h at 1 min intervals. Data were collected and analyzed using Microsoft Excel software. All assays were done in duplicate.

### 3.9. Statistics

The data are presented as means ± SEM and were analysed by the unpaired Student’s *t*-test using GraphPad Prism 8 software for Windows (GraphPad, San Diego, CA, USA), and those showing a *p*-value < 0.05 were considered significant.

## 4. Conclusions

We have shown that BBM stabilizes the TTR tetramer and in vitro and ex vivo approaches demonstrated that the binding occurs in the T4-binding pocket, with a capacity similar to IDIF, tafamidis and tolcapone, known as strong TTR stabilizers. A full characterization of the thermodynamic profile by ITC studies showed that the BBM enthalpically driven mode of action is through kinetic stabilization of TTR, with similar profiles to tafamidis and tolcapone.

The analysis of the crystal structure shows that BBM binds TTR in forward mode, with the bromine-substituted ring occupying the innermost part of the T4-binding site. BBM binding induces the rotation of the side residues of serine 117 of all four monomers, prompting the formation of strong intermonomer hydrogen bonds, which presumably increase the stability of the TTR tetramer.

Importantly, by a comparative kinetic analysis of the inhibition of TTR fibrillogenesis at moderately acid pH, we have concluded that BBM has similar inhibitory potency as known TTR stabilizer drugs.

On the basis of the obtained results, we can conclude that the uricosuric drug benzbromarone (BBM) presents an interesting scaffold in the quest to design of new and improved TTR stabilizers. Further studies are in progress to evaluate if this drug can be repurposed for FAP.

## Figures and Tables

**Table 1:** Thermodynamic parameters for interaction of TTR with small-molecule compounds including some TTR stabilizer drugs analyzed by ITC.

Assay	n	Kd (nM)	ΔG (Kcal/mol)	ΔH (Kcal/mol)	TΔS (Kcal/mol)
wtTTR + BBM	1	60	−9.93	−9.94	−0.02
wtTTR + IDIF	1	120	−9.49	−13.10	−3.61
wtTTR + tafamidis	2	200	−9.16	−6.50	2.66
wtTTR + tolcapone	2	270	−8.98	−12.30	−3.36
wtTTR + diflunisal	1	900	−8.25	−11.96	−3.70

## Supplementary Materials

Supplementary Materials can be found at https://www.mdpi.com/1422-0067/21/19/7166/s1. Data collection and refinement statistics; kinetics of aggregation of TTR in the presence of different stabilizers; list of organohalogen compounds among the TTR T_4_ tetramer stabilizers reported in PDB.

## Abbreviations

AD	Alzheimer’s disease
BBM	Benzbromarone
BSA	Bovine serum albumin
COMT	Catechol-*O*-methyltransferase
CSF	Cerebrospinal fluid
DIF	Diflunisal
DM	Dried milk
DMSO	Dimethyl sulfoxide
ECL	Enhanced chemiluminescence
EDTA	Ethylenediaminetetraacetic acid
FAP	Familial amyloid polyneuropathy
HBP	Halogen-binding pocket
HEPES	*N*-(2-Hydroxyethyl) piperazine-*N′*-(2-ethanesulfonic acid)
HPLC	High-performance liquid chromatography
IDIF	Iododiflunisal
ITC	Isothermal titration calorimetry
NMR	Nuclear magnetic resonance
NSAID	Non-steroidal anti-inflammatory drug
PBP	Pentabromophenol
PBS	Phosphate-buffered saline
PDB	Protein Data Bank
rpm	revolutions per minute
RT	Room temperature
SDS	Sodium dodecyl sulphate
TBBPA	Tetrabromobisphenol A
T4	Thyroxine
TBG	Thyroxine-binding globulin
TTR	Transthyretin
UPLC-TOF-MS	Ultra-high performance liquid chromatography time of flight mass spectrometry
