# Supplementary material for: Repurposing Benzbromarone for Familial Amyloid Polyneuropathy: A New Transthyretin Tetramer Stabilizer
Source: Int J Mol Sci. 2020 Sep 28;21(19):7166. doi: 10.3390/ijms21197166 (PMC7583827; doi:10.3390/ijms21197166)
Supplement: Supplementary file 1 [file ijms-21-07166-s001.pdf]

SUPPORTING INFORMATION

Repurposing benzbromarone for Familial Amyloid Polyneuropathy: a new transthyretin tetramer stabilizer.

Ellen Y. Cotrina,[1,†] Ângela Oliveira,[2,3,†] J.P. Leite, [2,3,4] Jordi Llop,[5] Luis Gales, [2,3,4] Jordi Quintana,[6] Isabel Cardoso,[*,2,3,4] and Gemma Arsequell.[*,1]

[1] Institut de Química Avançada de Catalunya (I.Q.A.C.-C.S.I.C.), 08034 Barcelona, Spain.
[2] IBMC - Instituto de Biologia Molecular e Celular, 4200-135 Porto, Portugal.
[3] i3S – Instituto de Investigação e Inovação em Saúde, Universidade do Porto, 4200-135 Porto, Portugal.
[4] Instituto de Ciências Biomédicas Abel Salazar (ICBAS), 4050-013 Porto, Portugal.
[5] CIC biomaGUNE, Basque Research and Technology Alliance (BRTA), 20014 San Sebastian, Spain.
[6] Research Programme on Biomedical Informatics, Universitat Pompeu Fabra (UPF-IMIM), 08003 Barcelona, Spain.

∗ Correspondence to:

Dr. Gemma Arsequell, e-mail: gemma.arsequell@iqac.csic.es

Dr. Isabel Cardoso, e-mail: icardoso@ibmc.up.pt

† These authors contributed equally to this work.

Table of contents	Page
Data collection and refinement statistics for the TTR:BBM complex	SI 3
Kinetic Turbidity Assay	SI 4
Time course of Y78F-hTTR fibril formation at pH 4.2, 37 °C in the presence of different concentrations of BBM	SI 4
Time course of Y78F-hTTR fibril formation at pH 4.2, 37 °C in the presence of different concentrations of Tafamidis<br><br>Time course of Y78F-hTTR fibril formation at pH 4.2, 37 °C in the presence of different concentrations of Tolcapone	SI 5
Time course of Y78F-hTTR fibril formation at pH 4.2, 37 °C in the presence of different concentrations of IDIF<br><br>Time course of Y78F-hTTR fibril formation at pH 4.2, 37 °C in the presence of different concentrations of Diflunisal	SI 6
Selected small-molecule ligands of transthyretin that share a common dibromophenol moiety (PDB)	SI 7

Table S1 – Data collection and refinement statistics for the TTR:BBM complex.

	TTR:BBM
**Data collection**	
Space Group	$P2_12_12$
Unit Cell dimensions	
a (Å)	42.9
b (Å)	85.1
c (Å)	64.4
α =β =γ (◦)	90
Resolution range (Å)	64.39 - 1.35
No. of observations (unique)	321843 (52367)
Multiplicity (overall/last shell)	6.1/ 5.5
Rmerge (%)* (overall/last shell)	7.1/ 106.8
Completeness (%)(overall/last shell)	100/ 100
I/s(I) (overall/last shell)	8.5/ 1.1
Mathews Coefficient ($Å^3Da^{-1}$)	2.18
Solvent content (%)	43.62
**Structure refinement**	
Rfactor† / Rfree (%)	17.7/ 21.5
No. of unique reflections (working / test set)	52298 (5158)
Water molecules	122
Total number of atoms	1979
Average B-factor ($Å^2$)	
Average protein B-factor ($Å^2$)	27.93
Average main-chain B-factor ($Å^2$)	23.70
Average side-chain B-factor ($Å^2$)	29.328
Average Benzbromarone B-factor ($Å^2$)	40.01
Average water B-factor ($Å^2$)	45.46
R.m.s. deviations from standard geometry	
Bonds (Å)	0.007
Angles (º)	0.96
Ramachandran plot statistics	
Most favoured regions (%)	97.79
Allowed regions (%)	2.21

* R merge =Σ|I –(I)|/ Σ(I), where I is the observed intensity and (I) is the average intensity of multiple observations of symmetry-related positions.
† R -factor= Σ $||/F_o|-|F_c||/\Sigma|F_o|$, where $|F_o|$ and $|F_c|$ are observed and calculated structure factor amplitudes respectively.

Kinetic Turbidity Assay:

Kinetics of aggregation of TTR in the presence of the following small-molecule compounds (TTR tetramer stabilizers)

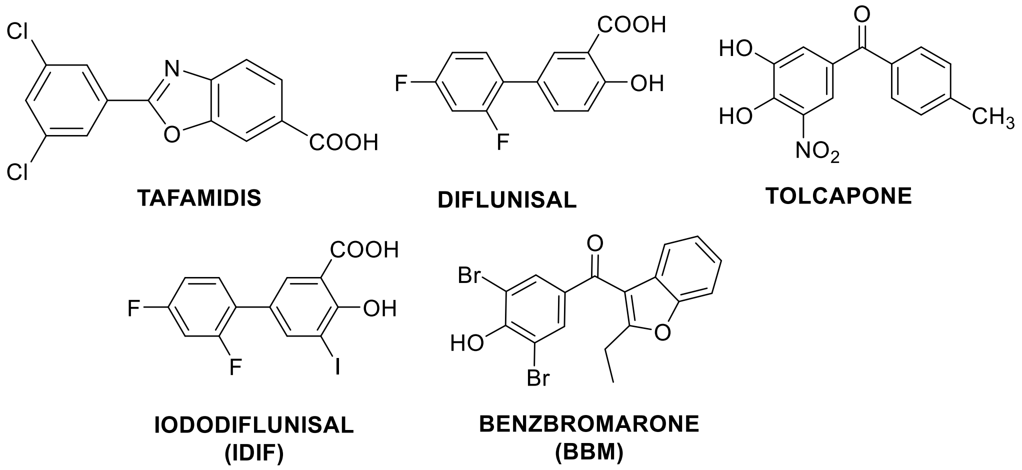

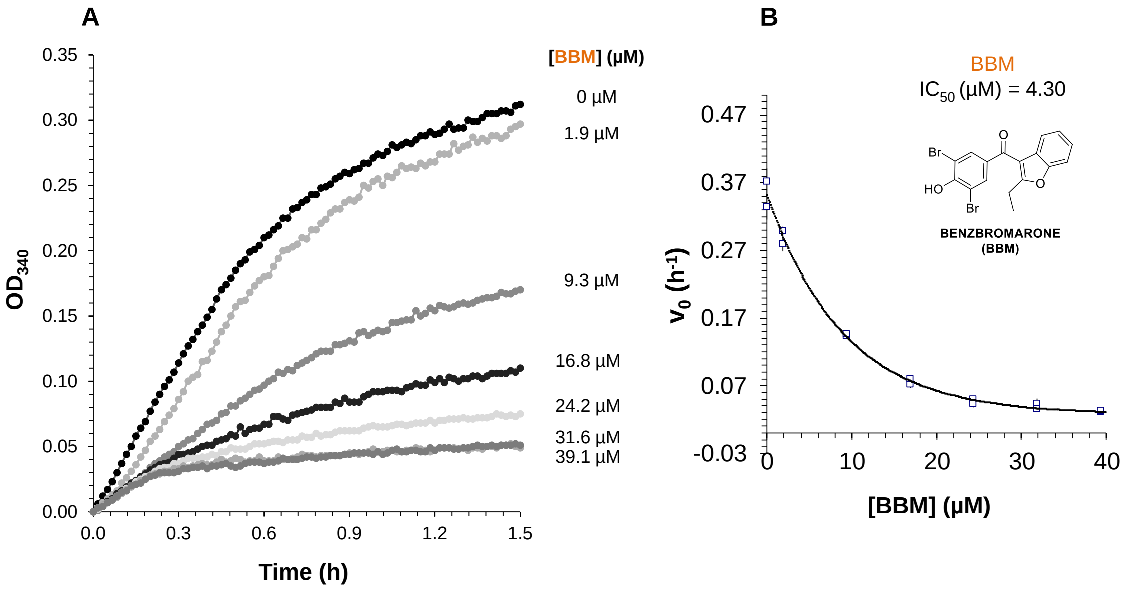

Figure S1. A) Time course of Y78F-hTTR fibril formation at pH 4.2, 37 °C in the presence of different concentrations of BBM. B) Plot of initial rates of fibril formation (V_0) vs BBM concentration (Data were fitted to eq 1); as monitored by absorbance at 340 nm at different concentrations.

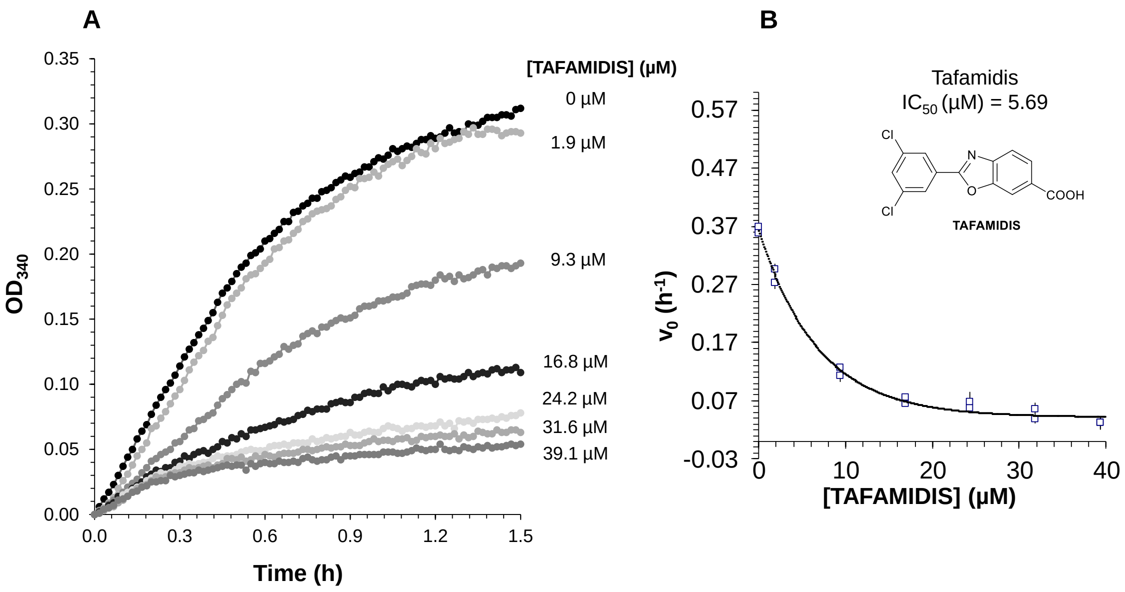

Figure S2. A) Time course of Y78F-hTTR fibril formation at pH 4.2, 37 °C in the presence of different concentrations of Tafamidis. B) Plot of initial rates of fibril formation (V_0) vs Tafamidis concentration (Data were fitted to eq 1); as monitored by absorbance at 340 nm at different concentrations.

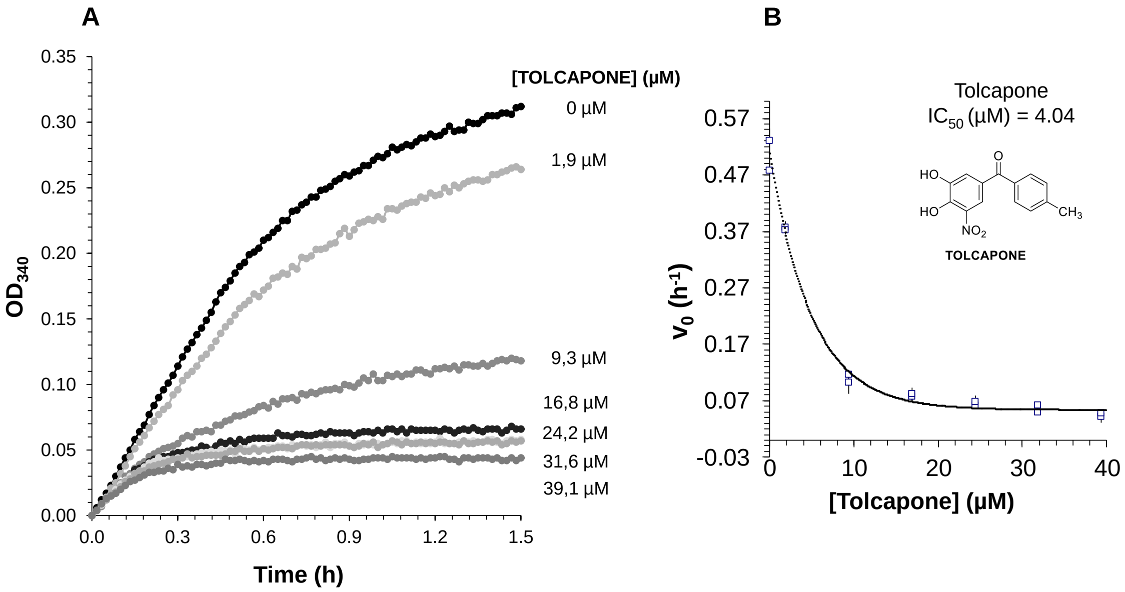

Figure S3. A) Time course of Y78F-hTTR fibril formation at pH 4.2, 37 °C in the presence of different concentrations of Tolcapone. B) Plot of initial rates of fibril formation (V_0) vs Tocalpone concentration (Data were fitted to eq 1); as monitored by absorbance at 340 nm at different concentrations.

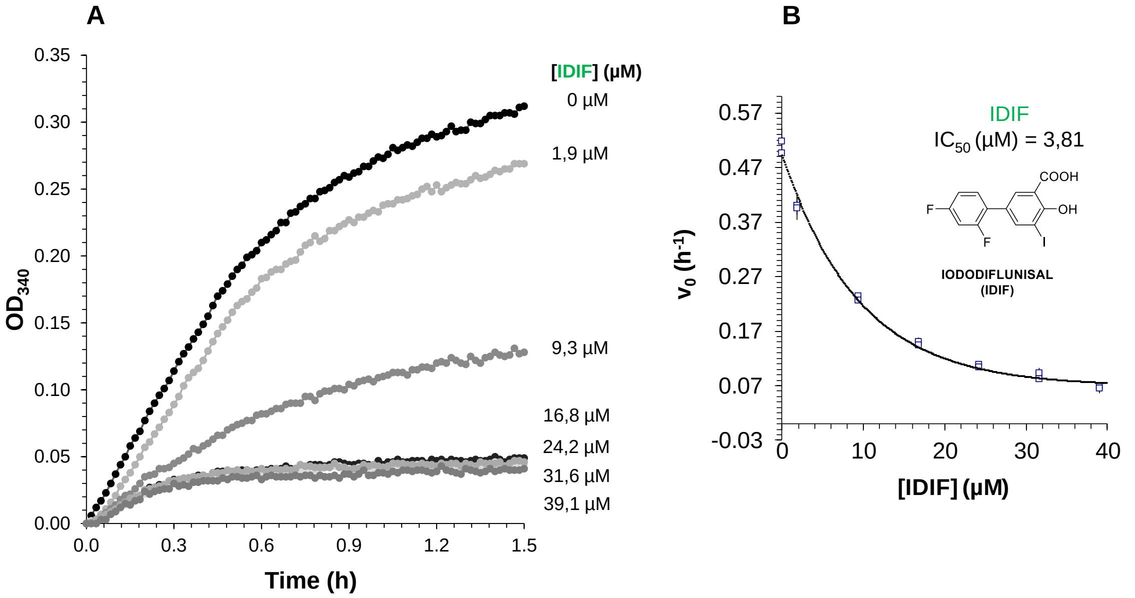

Figure S4. A) Time course of Y78F-hTTR fibril formation at pH 4.2, 37 °C in the presence of different concentrations of Iododiflunisal (IDIF). B) Plot of initial rates of fibril formation (V_0) vs IDIF concentration (Data were fitted to eq 1); as monitored by absorbance at 340 nm at different concentrations.

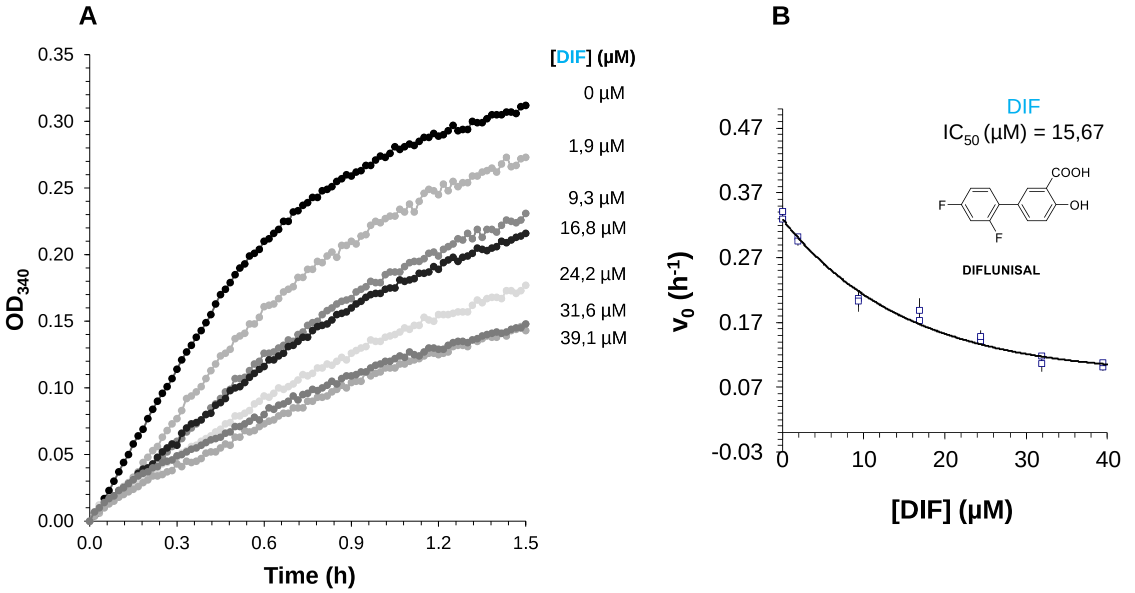

Figure S5. A) Time course of Y78F-hTTR fibril formation at pH 4.2, 37 °C in the presence of different concentrations of Diflunisal (DIF). B) Plot of initial rates of fibril formation (V_0) vs DIF concentration (Data were fitted to eq 1); as monitored by absorbance at 340 nm at different concentrations.

Table S2: Information extracted form the pdb on selected small-molecule ligands of transthyretin that share a common dibromophenol moiety.

PDB ID	Ligand ID	Structure	Ligand Formula	Ligand MW	Ligand Name	Title	Date released	DOI
1E4H	PBR	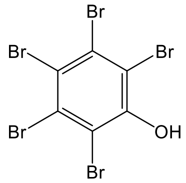	C6 H BR5 O	488.59	PENTABROMOPH ENOL	STRUCTURE OF HUMAN TRANSTHYRETIN COMPLEXED WITH BROMOPHENOLS-A NEW MODE OF BINDING	29/08/2000	10.2210/pdb1e4h/pdb
1E5A	TBP	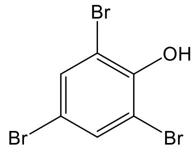	C6 H3 BR3 O	330.80	2,4,6-TRIBROMOPHENO L	STRUCTURE OF HUMAN TRANSTHYRETIN COMPLEXED WITH BROMOPHENOLS-A NEW MODE OF BINDING	30/08/2000	10.2210/pdb1e4h/pdb
1KGJ	FL8	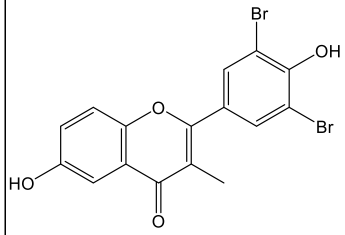	C16 H10 BR2 O4	426.06	6,4'-DIHYDROXY-3-METHYL-3',5'-DIBROMOFLAVON E	RAT TRANSTHYRETIN (ALSO CALLED PREALBUMIN) COMPLEX WITH 3',5' -DIBROMOFLAVONE (EMD21388)	27/11/2002	10.2210/pdb1kgj/pdb
1THC	FL9	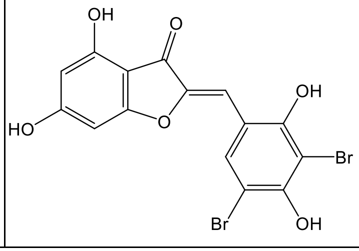	C15 H8 BR2 O6	444.03	3',5'-DIBROMO-2',4,4',6'-TETRAHYDROXY AURONE	CRYSTAL STRUCTURE DETERMINATION AT 2.3 A OF HUMAN TRANSTHYRETIN 3', 5' -DIBROMO -2', 4, 4', 6 -TETRA-HYDROXYAURONE COMPLEX	15/07/1993	10.2210/pdb1thc/pdb

2QGD	MR5	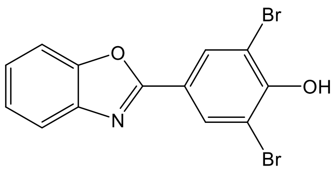	C13 H7 BR2 N O2	369.01	4-(1,3-BENZOXAZOL-2-YL)-2,6-DIBROMOPHENOL	HUMAN TRANSTHYRETIN (TTR) COMPLEXED WITH 2-(3, 5-DIBROMO-4-HYDROXYPHENYL) BENZOXAZOLE	05/02/2008	10.2210/pdb2qgd/pdb
3CN1	LJ2	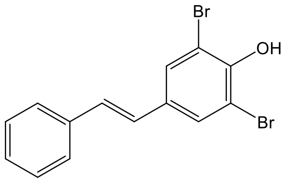	C14 H10 BR2 O	354.04	2,6-DIBROMO-4-[(E)-2-PHENYLETHENYL] PHENOL	HUMAN TRANSTHYRETIN (TTR) IN COMPLEX WITH 3, 5-DIBROMO-4-HYDROXYSTILBENE	28/10/2008	10.2210/pdb3cn1/pdb
3CN2	LJ3	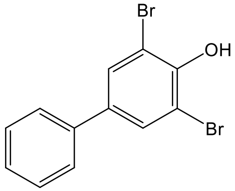	C12 H8 BR2 O	328.00	3,5-DIBROMOBIPHENYL-4-OL	HUMAN TRANSTHYRETIN (TTR) IN COMPLEX WITH 3, 5-DIBROMO-4-HYDROXYBYPHENYL	28/10/2008	10.2210/pdb3cn2/pdb
3CN3	LJ4	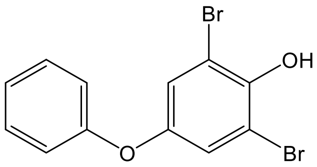	C12 H8 BR2 O2	344.00	2,6-DIBROMO-4-PHENOXYPHENOL	HUMAN TRANSTHYRETIN (TTR) IN COMPLEX WITH 1, 3-DIBROMO-2-HYDROXY-5-PHENOXYBENZENE	28/10/2008	10.2210/pdb3cn3/pdb
3CN4	LJ5	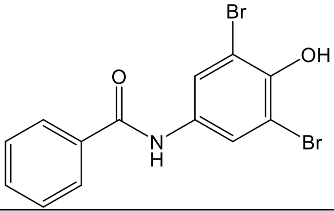	C13 H9 BR2 N O2	371.03	N-(3,5-DIBROMO-4-HYDROXYPHENYL)BENZAMIDE	HUMAN TRANSTHYRETIN (TTR) IN COMPLEX WITH N-(3, 5-DIBROMO-4-HYDROXYPHENYL)BENZAMIDE	28/10/2008	10.2210/pdb3cn4/pdb
3ESN	DZ1	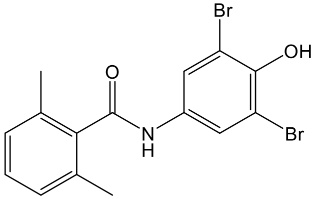	C15 H13 BR2 N O2	399.08	N-(3,5-DIBROMO-4-HYDROXYPHENYL)-2,6-DIMETHYLBENZAMIDE	HUMAN TRANSTHYRETIN (TTR) COMPLEXED WITH N-(3, 5-DIBROMO-4-HYDROXYPHENYL)-2,6-DIMETHYLBENZAMIDE	07/04/2009	10.2210/pdb3esn/pdb

3ESO	DZ2	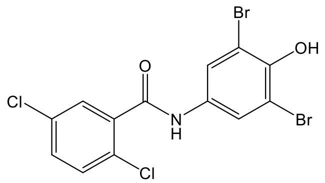	C13 H7 BR2 CL2 N O2	439.92	2,5-DICHLORO-N-(3,5-DIBROMO-4-HYDROXYPHENYL)BENZAMIDE	HUMAN TRANSTHYRETIN (TTR) COMPLEXED WITH N-(3, 5-DIBROMO-4-HYDROXYPHENYL)-2,6-DICHLOROBENZAMIDE	07/04/2009	10.2210/pdb3eso/pdb
3ESP	DZ3	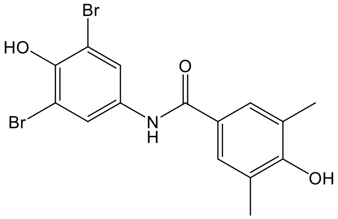	C15 H13 BR2 N O3	415.08	N-(3,5-DIBROMO-4-HYDROXYPHENYL)-4-HYDROXY-3,5-DIMETHYLBENZAMIDE	HUMAN TRANSTHYRETIN (TTR) COMPLEXED WITH N-(3, 5-DIBROMO-4-HYDROXYPHENYL)-3, 5-DIMETHYL-4-HYDROXYBENZAMIDE	07/04/2009	10.2210/pdb3esp/pdb
3IMR	IW1	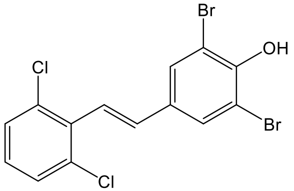	C14 H8 BR2 CL2 O	422.93	2,6-DIBROMO-4-[(E)-2-(2,6-DICHLOROPHENYL)ETHENYL]PHENOL	TRANSTHYRETIN IN COMPLEX WITH (E)-2,6-DIBROMO-4-(2,6-DICHLOROSTYRYL)PHENOL	12/01/2010	10.2210/pdb3imr/pdb
3IMS	IW2	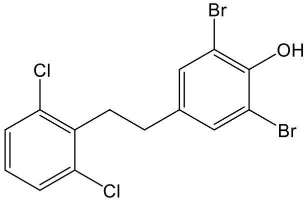	C14 H10 BR2 CL2 O	424.95	2,6-DIBROMO-4-[2-(2,6-DICHLOROPHENYL)ETHYL]PHENOL	TRANSTHYRETIN IN COMPLEX WITH 2,6-DIBROMO-4-(2,6-DICHLOROPHENETHYL)PHENOL	12/01/2010	10.2210/pdb3ims/pdb
3IMT	IW3	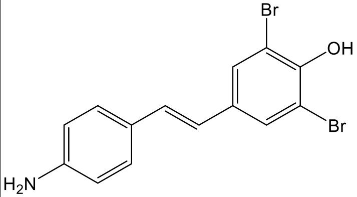	C14 H11 BR2 N O	369.05	4-[(E)-2-(4-AMINOPHENYL)ETHENYL]-2,6-DIBROMOPHENOL	TRANSTHYRETIN IN COMPLEX WITH (E)-4-(4-AMINOSTYRYL)-2,6-DIBROMOPHENOL	12/01/2010	10.2210/pdb3imt/pdb
3IMU	IW4	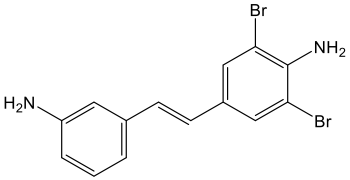	C14 H12 BR2 N2	368.07	4-[(E)-2-(3-AMINOPHENYL)ETHENYL]-2,6-DIBROMOANILINE	TRANSTHYRETIN IN COMPLEX WITH (E)-4-(3-AMINOSTYRYL)-2,6-DIBROMOANILINE	12/01/2010	10.2210/pdb3imu/pdb

**3IMV**	**IW5**	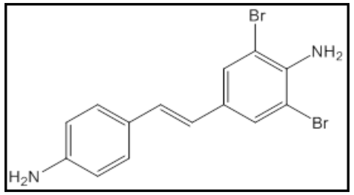	C14 H12 BR2 N2	368.07	4-[(E)-2-(4-AMINOPHENYL)ETHENYL]-2,6-DIBROMOANILINE	TRANSTHYRETIN IN COMPLEX WITH (E)-4-(4-AMINOSTYRYL)-2,6-DIBROMOANILINE	12/01/2010	10.2210/pdb3imv/pdb
**3IMW**	**IW6**	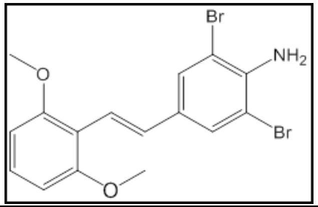	C16 H15 BR2 N O2	413.11	2,6-DIBROMO-4-[(E)-2-(2,6-DIMETHOXYPHENYL)ETHENYL]ANILINE	TRANSTHYRETIN IN COMPLEX WITH (E)-2,6-DIBROMO-4-(2,6-DIMETHOXYSTYRYL)ANILINE	12/01/2010	10.2210/pdb3imw/pdb
**3P3S**	**3M2**	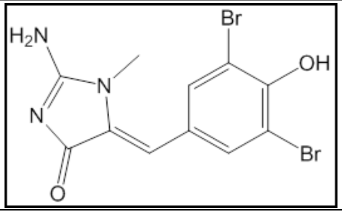	C11 H9 BR2 N3 O2	375.02	(5Z)-2-AMINO-5-(3,5-DIBROMO-4-HYDROXYBENZYLIDENE)-1-METHYL-1,5-DIHYDRO-4H-IMIDAZOL-4-ONE	HUMAN TRANSTHYRETIN (TTR) COMPLEXED WITH (Z)-5-(3,5-DIBROMO-4-HYDROXYBENZYLIDENE)-IMINO-1-METHYLIMIDAZOLIDIN-4-ONE	24/08/2011	10.2210/pdb3p3s/pdb
**4FI8**	**0UC**	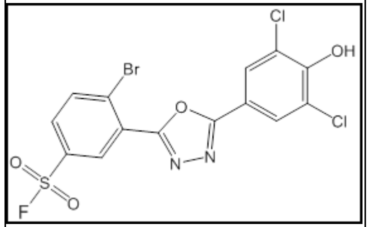	C14 H6 BR CL2 F N2 O4 S	468.08	4-BROMO-3-[5-(3,5-DICHLORO-4-HYDROXYPHENYL)-1,3,4-OXADIAZOL-2-YL]BENZENESULFONYL FLUORIDE	KINETIC STABILIZATION OF TRANSTHYRETIN THROUGH COVALENT MODIFICATION OF K15 BY 4-BROMO-3-(5-(3,5-DICHLORO-4-HYDROXYPHENYL)-1,3,4-OXADIAZOL-2-YL)-BENZENESULFONAMIDE	20/02/2013	10.2210/pdb4fi8/pdb
**4PM1**	**ESZ**	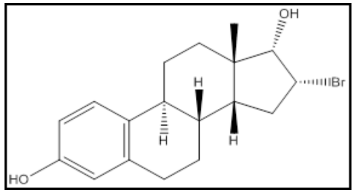	C18 H23 Br O2	351.28	(14beta,16alpha,17alpha)-16-BROMOESTRA-1,3,5(10)-TRIENE-3,17-DIOL	HUMAN TRANSTHYRETIN (TTR) COMPLEXED WITH 16-ALPHA-BROMO-ESTRADIOL	08/10/2014	10.2210/pdb4pm1/pdb

5E23	L32	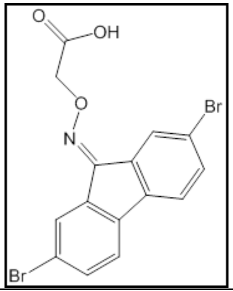	C15 H9 Br2 N O3	411.05	{[(2,7-DIBROMO-9H-FLUOREN-9-YLIDENE)AMINO]OXY}ACETIC ACID	HUMAN TRANSTHYRETIN (TTR) COMPLEXED WITH (2,7-DIBROMO-FLUOREN-9-YLIDENEAMINOOXY)-ACETIC ACID	23/06/2016	10.2210/pdb5e23/pdb
5HJG	XDI	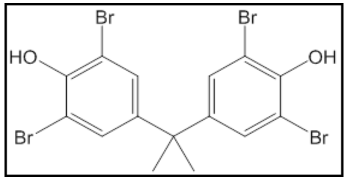	C15 H12 Br4 O2	543.87	4,4'-PROPANE-2,2-DIYBIS(2,6-DIBROMOPHENOL)	CRYSTAL STRUCTURE OF HUMAN TRANSTHYRETIN IN COMPLEX WITH TETRABROMOBISPHENOL A (TBBPA)	04/05/2016	10.2210/pdb5hjg/pdb
